# Ethical, legal, organizational and social issues related to the use of scalp cooling for the prevention of chemotherapy‐induced alopecia: A systematic review

**DOI:** 10.1111/hex.13679

**Published:** 2022-12-30

**Authors:** Janet Delgado Rodríguez, Vanesa Ramos‐García, Diego Infante‐Ventura, José Carlos Suarez‐Herrera, Antonio Rueda‐Domínguez, Pedro Serrano‐Aguilar, María del Mar Trujillo‐Martín

**Affiliations:** ^1^ Department of Philosophy University of Granada Granada Spain; ^2^ The Spanish Network of Agencies for Health Technology Assessment and Services of the National Health System (RedETS) Tenerife Spain; ^3^ Canary Islands Health Research Institute Foundation (FIISC) Tenerife Spain; ^4^ Network for Research on Chronicity Primary Care, and Health Promotion (RICAPPS) Tenerife Spain; ^5^ Department of Strategy Entrepreneurship and Sustainable Development KEDGE Business School Marseille France; ^6^ Cátedra UNITWIN/UNESCO de IPD‐SILOS Universidad de Las Palmas de Gran Canaria Las Palmas Spain; ^7^ Medical Oncology Intercenter Unit Regional and Virgen de la Victoria University Hospitals, IBIMA Málaga Spain; ^8^ Research Network on Health Services in Chronic Diseases (REDISSEC) Málaga Spain; ^9^ Evaluation Unit of the Canary Islands Health Service (SESCS) Tenerife Spain; ^10^ Research Network on Health Services in Chronic Diseases (REDISSEC) Tenerife Spain

**Keywords:** chemotherapy‐induced alopecia, ethical, healthcare professional perspective, legal, organizational and social issues

## Abstract

**Introduction:**

Scalp cooling (SC) aims to prevent chemotherapy‐induced alopecia. The goal of this systematic review is to tackle ethical, legal, organizational and social issues related to SC.

**Methods:**

A critical appraisal of the literature was carried out using a systematic review design. MEDLINE, Embase and Web of Science databases were searched up until 2 June 2021. Studies addressing these aspects in English or Spanish were considered. Representatives of both patient associations and professional scientific societies related to the topic participated in the design of the protocol and the review of the findings.

**Results:**

A total of 17 studies were included. Articles were critically appraised using the MMAT and SANRA. Findings were organized into four categories: (1) ethical aspects focused on equal access, gender equity and doctor–patient communication supported by Patient Decision Aids (PtDAs); (2) patient perspective and acceptability; (3) professional perspective and acceptability; (4) organizational aspects focused on accessibility and feasibility.

**Conclusion:**

Cancer patients' expectations when using SC need to be adjusted to reduce the potential distress associated with hair loss. PtDAs could help patients clarify their values and preferences regarding SC. Equal access to technology should be guaranteed.

**Patient or Public Contribution:**

In this systematic review, the representatives of the patient associations (Ms. María Luz Amador Muñoz of the Spanish Association Against Cancer [AECC] and Ms. Catiana Martinez Cánovas of the Spanish Breast Cancer Federation [FECMA]) participated in the review of the study protocol, as well as in the results, discussion and conclusions, making their contributions. In the type of design of these studies (systematic reviews), it is not usual to have the direct participation of patients, but in this one, we have done so, as it is a systematic review that is part of a report of the Spanish Network of Health Technology Assessment Agencies (ETS).

## 1 INTRODUCTION

Alopecia is one of the most common and visible adverse effects of chemotherapy, which affects approximately 65% of all patients undergoing chemotherapy.^1^, ^2^

Although chemotherapy‐induced alopecia (CIA) is not life‐threatening and in most cases is reversible, it can have a significant impact on a patient's quality of life, especially in psychological and social terms.^3^, ^4^ While it has long been considered an acceptable side effect in the treatment of patients, the increasing number of cancer survivors and a better understanding of the associated psychological processes have led to a greater consideration of CIA as a relevant problem.^5^ The negative psychosocial effects associated with CIA are strongly related to the diversity of sociocultural values and symbolic assignments attributed to hair.^6^, ^7^, ^8^ As an essential element in personal identity, hair loss causes high levels of stress and anxiety, and makes it difficult to perform daily activities, especially those in social contexts.^9^ The clear visibility of alopecia for patients and the people around them acts to identify the cancer and usually, when alopecia appears it becomes the moment of public recognition of the disease.^9^, ^10^ As a result, people with CIA may begin to perceive certain changes in the attitudes towards them of the people they relate to, ranging from sympathy to rejection.^11^, ^12^

Scalp cooling (SC) has been used since the late 1970s as a system to prevent CIA.^13^ Reduction in scalp temperature induces vasoconstriction, which limits the arrival of chemotherapeutic agents to the scalp and also produces a reduction in metabolism in the cells present in hair follicles at the time of highest chemotherapeutic concentration in the blood plasma. This reduces their vulnerability to the antimitotic and antimetabolic effects of these drugs.^14^ The scalp must attain a subcutaneous temperature (between 1 and 2 mm) below 22°C,^15^ which is equivalent to an epicutaneous temperature of 19°C,^14^ although greater preventive effects could be achieved with temperatures close to 15°C.^16^ A correct adjustment of the SC system to the patient's head is essential to attain these temperatures consistently and homogeneously.^17^, ^18^ The SC activation should commence 5 and 30 min before the infusion of cytostatic drugs and continue until completion.^18^ After completion of the infusion, the SC must remain for a more or less prolonged time depending on the pharmacokinetics of the chemotherapeutic agents used, with postinfusion cooling times varying from 15 min to 4 h.^18^

In 2021, the Spanish Network of Agencies for Health Technology Assessment (HTA) and Services of the National Health System (RedETS) drew up an HTA report^19^ commissioned by the Spanish Ministry of Health on the effectiveness, safety and cost‐effectiveness of SC devices for the prevention of CIA. This Systematic Review (SR) was conducted as part of this HTA report. This report commissioned by the Ministry of Health of our country has the main objective of informing the political decision to include the technology in the common portfolio of health services of the National Health System, but it is also useful in decision‐making for clinicians and patients.^20^ Particularly, the ethical, legal, organizational and social issues are relevant when considering the equity in access, feasibility and acceptability of the technology, which are key elements to take into account for a successful implementation of the technology.

The research questions were: what are the ethical and legal implications of the use of SC? What are the attitudes, perceptions and experiences of patients and healthcare professionals regarding SC systems to prevent CIA? Are there any organizational aspects that may affect the accessibility or feasibility of the SC?

## 2 METHODS

### 2.1 Protocol and registration

The SR of the literature followed the methodologic guidelines drawn up by the Cochrane Collaboration^21^ with reporting in accordance with the Preferred Reporting Items for Systematic Reviews and Meta‐Analyses (PRISMA) statement. The prespecified protocol for this review was registered in PROSPERO (registration number CRD42021268228). The PRISMA checklist is available in Supporting Information: File 1.

### 2.2 Design and participation of stakeholders

In this SR, the representatives of patient associations such as the Spanish Association Against Cancer (AECC) and the Spanish Breast Cancer Federation (FECMA) participated in the review of the study protocol, as well as in the results, discussion and conclusions, having the opportunity to make their contributions. In addition, representatives of professional scientific societies related to the topic such as the Spanish Society of Medical Oncology (SEOM) were invited to participate. Although direct patient participation is not common in systematic reviews, we have integrated it into this study because we believe that the involvement of patients and other stakeholders in the research process is crucia.^22^

### 2.3 Search strategy

The electronic databases MEDLINE (using the Ovid platform), EMBASE (Elsevier interface) and WOS were searched from database inception to June 2, 2021. The search strategy included both controlled vocabulary and text‐word terms related to ‘chemotherapy’, ‘alopecia’ and ‘hair loss’. The search was restricted to studies published in English or Spanish. The full search strategy used for each database is available in Supporting Information: File 2.

### 2.4 Selection criteria

Studies were eligible for inclusion if they fulfilled the criteria shown in Table 1.

**Table 1:** Study selection criteria

Design	Experimental, quasi‐experimental, observational, qualitative or mixed‐methods studies, systematic and narrative reviews and theoretical articles
Population	Adults (>18 years) with cancer eligible to receive, who are receiving or have received intravenous chemotherapy treatment and/or healthcare professionals using SC technology
Intervention	Application of the SC system
Outcome	Ethical aspects (patient values, morals, culture and autonomy, risk benefit ratio, human rights, dignity), legal aspects (laws, regulations, data protection, human rights, property and responsibility, market regulation), organizational aspects (process or work flow, planning or implementation, informational and training needs, acceptability) and social aspects (impact, perspective of patients and caregivers, experiences and preferences with cancer and the use of SC, important outcome measures for patients, self‐management, information and support needs, acceptability)
Language	English or Spanish
Date of publication	No restrictions

### 2.5 Study selection

Two reviewers independently screened and in duplicate the title and abstract of retrieved references to identify potentially eligible studies. The full text of these references was then screened again in duplicate to confirm eligibility. Doubts and discrepancies between reviewers were resolved by discussion and, when no consensus was reached, a third reviewer was consulted.

### 2.6 Data extraction and quality assessment

Data were extracted from studies included by one reviewer and checked by a second reviewer using a piloted form in Excel format devised by the authors that included the following items: general information (authors, publication year, country and funding), study design, population, measures used, main findings, study limitations and conflict of interest.

Two reviewers independently and in duplicate assessed possible methodologic limitations of the studies included. We planned to assess quantitative, qualitative and mixed methods studies using the Mixed Methods Assessment Tool (MMAT).^23^ The quality of systematic reviews and narrative reviews were assessed with the aid of the AMSTAR‐2^24^ and SANRA tools,^25^ respectively. Descriptive studies, qualitative studies and mixed methods studies were then assessed using MMAT.^23^ This tool permits to appraise the methodological quality of five categories of studies: qualitative research, randomized controlled trials, non‐randomized studies, quantitative descriptive studies and mixed methods studies. For each relevant study, we used the corresponding criteria to appraise the study's quality, conduct the appraisal process and determined an overall quality score for each study. In the case of SANRA, used for the assessment of narrative articles, although the authors of the tool do not establish cut‐offs for different grades of quality, we considered a score of ≤5 as low, 6–8 as moderate and 9–12 as high quality.

### 2.7 Data synthesis

A narrative synthesis of the main themes found was performed, taking into account criteria of relevance (applicability to the context specified in the review question), coherence (how clear and well supported are the data from the primary studies and from the synthesized outcomes provided by reviews) and adequacy (the degree of richness and amount of data supporting a review conclusion).^23^, ^26^

## 3 RESULTS

**Figure 1:**
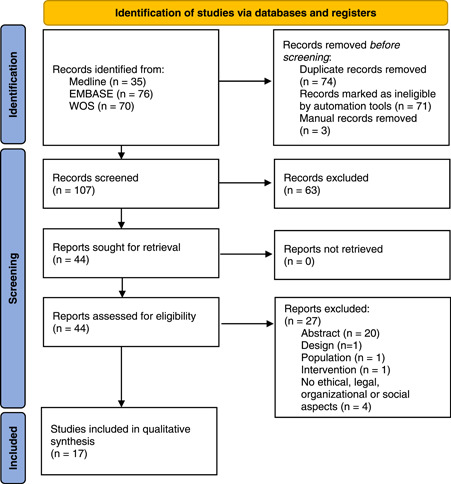
PRISMA 2020 flow diagram for systematic reviews. *Source*: Page et al.^27^

From a total of 107 records initially identified, after eliminating duplicates, through the database search, 44 potentially relevant publications were selected after the title and abstract screening, of which 17 studies were finally eligible for inclusion according to the pre‐established selection criteria (Figure 1). Table 2 shows the main characteristics and overall quality of the studies included. Detailed quality assessments of the studies are available in Supporting Information: File 3. The studies were published between 1996 and 2020, all in English. Five are descriptive studies,^28^, ^29^, ^30^, ^31^, ^32^ four narrative reviews,^33^, ^34^, ^35^, ^36^ three qualitative studies,^37^, ^38^, ^39^ two mixed methods studies,^40^, ^41^ two theoretical articles about implementation experience^42^, ^43^ and one observational study.^44^

**Table 2:** Main characteristics of studies included

References	Objective	Methods	Population (*N*)	Quality
Bitto et al.^28^	To assess the effects of the SC system on quality of life in patients with BC	Descriptive study using a questionnaire	BC patients (70)	High^a^
Breed et al.^33^	Raise awareness of the impact of CIA to demonstrate the potential for prevention of CIA by SC	Narrative review	Patients undergoing chemotherapy	9 (High)^b^
Dougherty^37^	Know the opinion of patients about hair loss and SC	Qualitative study through semistructured interviews	Cancer patients undergoing intravenous chemotherapy	Low^a^
Fischer‐Cartlidge et al.^42^	Describe the experience of a large multisite organization that implemented an SC programme	Theoretical article: Implementation experience	Patients undergoing chemotherapy	NE
Heery et al.^43^	Describe the implementation of a SC programme in a community breast health centre	Theoretical article: Implementation experience	Patients with BC undergoing chemotherapy, users of DIGNICAP® (67) and PENGUIN^TM^ (25). Patients with other types of cancer users of both systems (14)	NE
Lemieux et al.^29^	To know the opinion of health professionals on the use of SC in the reduction of alopecia in patients with BC and participation in an RCT	Descriptive study using a questionnaire	Patients with BC undergoing chemotherapy	Low^a^
Massey^44^	To assess the efficacy of SC technology in patients receiving treatment for BC and to assess their views about its comfort and acceptability	Observational study	Patients with BC undergoing chemotherapy (94)	Low^a^
Mols et al.^30^	To explore the attitudes of the nursing staff on ECC technology and the perception and opinion of alopecia	Descriptive study using a questionnaire	Patients with BC undergoing SC (98) and without the technology (168)	Low^a^
Peerbooms et al.^31^	To know the familiarity, opinions and attitudes of patients and oncology professionals about SC technology	Descriptive study using a questionnaire	Nursing professionals (49) Oncology professionals (100) Former cancer patients (177)	Low^a^
Peterson et al.^34^	To present a pragmatic workflow for collaborative efforts of healthcare professionals to provide SC in patients undergoing treatment regimens known to affect hair follicles	Narrative review	Patients undergoing chemotherapy	8 (moderate)^b^
Randall and Ream^40^	To explore the attitudes of the nursing staff on technology and the perception and opinion of alopecia	Mixed methods study (questionnaires and interviews)	Nursing staff Questionnaire (13) and interviews (3)	Low^a^
Roe^35^	To explore the issue of CIA from the patient's perspective and SC as a preventive measure, along with a review of the evidence on the risk associated with the development of scalp metastases after SC	Narrative review and discussion article	Patients undergoing chemotherapy	9 (high)^b^
Shaw et al.^38^	To explore patients' perceptions and experience on SC	Qualitative study through focus groups and interviews	Patients with BC undergoing chemotherapy, both users of cooling systems and not (17)	High^a^
Shaw et al.^39^	To qualitatively explore healthcare professionals' perceptions, barriers and facilitators to the implementation of SC in Australian cancer centres	Qualitative study using telephone interviews	Health professionals working in oncology (21) in five cancer centres, in some of which SC therapy was performed and in others it was not	High^a^
Van Den Hurk et al.^32^	To explore the severity and burden of hair loss in cancer patients treated with chemotherapy, level of satisfaction with wigs, hair growth and body image	Descriptive study using a questionnaire	Patients with BC undergoing SC (98) and without the technology (168)	Low^a^
Van Den Hurk et al.^41^	Provide up‐to‐date online information on CIA and SC to help patients cope with CIA and their choice of SC	Mixed methods study	Focus groups: patients with BC with CIA (9) and patients with BC who received SC (6). Semistructured interviews: SC‐eligible BC patients (11). Questionnaires: nursing staff (10)	Low^a^
Young and Arif^36^	To explore clinicians' barriers to the application of SC as well as to present data on recent studies	Narrative review	Medical and nursing professionals	6 (moderate)^b^

Abbreviations: BC, breast cancer; CIA, chemotherapy‐induced alopecia; NE, not evaluated; RCT, randomized controlled trial; SC, scalp cooling.

^a^ Assessed with MMAT (Mixed Methods Assessment Tool).

^b^ Assessed with SANRA (scale for the quality assessment of narrative review article).

The studies included in the review were synthesized according to their relevance, coherence and adequacy, and different standard tools were used to assess the quality of the studies, depending on their design (Table 2). All the quality assessments of the studies are available in Supporting Information: File 3. We found that the quality of four of the five descriptive studies^29^, ^30^, ^31^, ^32^ was considered low, and only the study by Bitto et al.^28^ had high quality. Aspects evaluated with this tool were the sampling strategy, the representativeness, if the measurements are appropriate, the risk of nonresponse bias and if the statistical analysis is appropriate. Although only three qualitative studies, the quality of two of them was considered high^38^, ^39^ and one^37^ low. The aspects assessed for qualitative studies were the adequacy of the qualitative approach, the method of data collection if the findings are adequately derived from the data, the appropriate interpretation of results and the coherence. Two mixed‐methods studies were also found,^40^, ^41^ but the quality was rated as low, the same as the observational study included.^44^ The aspects assessed were the adequacy of the rationale, the integration of the different components of the study, whether the outputs were adequately interpreted, the divergences and inconsistencies between quantitative and qualitative results, and if the quality criteria of each method were followed. The SANRA tool^25^ was used to evaluate the quality of narrative reviews, and thus the four narrative reviews included were of a moderate‐high quality.^33^, ^34^, ^35^, ^36^ With this tool aspects such as justification, explicit aims, description of the literature search, referencing, scientific reasoning and appropriate presentation of data were assessed.

A thematic analysis of the review results was performed and the main findings were categorized according to four relevant dimensions: (1) ethical aspects focused on equal access, gender equity and doctor–patient communication supported by Patient Decision Aids (PtDAs); (2) patient perspective and acceptability; (3) professional perspective and acceptability; (4) organizational aspects focused on accessibility and feasibility. No results were found on legal aspects. A summary of the findings is shown in Table 3.

**Table 3:** Main findings

Finding	Studies that contribute to the findings
Ethical aspects focused on equal access, gender equity and doctor–patient communication supported by Patient Decision Aids (PtDAs)	
Health professionals should learn when to offer SC according to the characteristics of each patient. Patient education should include expected results, normal hair loss that may occur and proper care and precautions for hair. Professionals should consider possible causes of distress for their patients.	Dougherty,^37^ Roe,^35^ Peterson et al.^34^
Patient experiences influence oncology healthcare professionals' attitudes towards SC technology.	Shaw et al.^39^
Online decision‐making tools, scientific information and practical advice on CIA and SC have been developed, as well as a value clarification exercise. https://www.scalpcooling.org/ (last access 11 August 2021).	Van Den Hurk et al.^41^
Patient perspective and acceptability	
Patients who undergo SC may have a mismatch between their expectations and their experience, as well as feel greater distress if their hair begins to fall out despite SC therapy, compared to those who do not undergo SC, since that for the latter, alopecia is an expected result of chemotherapy.	Breed et al.,^33^ Shaw et al.^38^
Factors favoring acceptability are faster hair growth, the attitude of the nursing staff towards the SC.	Shaw et al.,^38^ Shaw et al.^39^
Barriers to acceptability are the fact that the SC technology does not guarantee that the hair will be kept, having to spend more time in the hospital, the potential risk of skin metastases to the scalp, thinking that it may be too cold, inability to of tolerating the cold cap for 5 h, prioritizing hair colouring to mask grey, finding using colour powder for touch‐ups too time‐consuming or finding that washing hair only once a week was intolerable.	Peerbooms et al.,^31^ Van Den Hurk et al.,^41^ Heery et al.^43^
Professional perspective and acceptability	
Health professionals should discuss SC with both men and women in a way that enables men to discuss their concerns. They should not assume that men do not have concerns about hair loss, as they may also want to keep their hair during chemotherapy.	Randall and Ream,^40^ Breed et al.,^33^ Roe^35^
Nursing staff may be charged with informing their patients about the SC to provide them with sufficient knowledge about the risks and benefits, to make an informed decision.	Young and Arif^36^
Although oncology professionals consider the implementation of SC technology in their chemotherapy unit acceptable, they do not consider it feasible for patients to remain in the ward 90 min after the chemotherapy session to end the SC therapy.	Lemieux et al.^29^
Approximately 50% of oncology professionals consider the implementation of SC technology in their chemotherapy unit to be acceptable.	Lemieux et al.^29^
Some facilitators for the acceptability of the professionals include considering it as a service for their patients, the fact that patients actively request it, the participation of personnel in the decision‐making for implementation and the commitment between the medical and nursing staff. Attitudes towards the need for intervention on alopecia and towards SC influenced professionals to defend the technology within their centres and offer the treatment to their patients.	Peerbooms et al.,^31^ Shaw et al.^39^
Some barriers are the lack of evidence on efficacy and safety, little evidence about the risk of metastases to the scalp skin, logistic difficulties and lack of organizational support.	Peerbooms et al.,^31^ Shaw et al.,^39^ Fischer‐Cartlidge et al.^42^
Professionals perceive that SC supposes an increased workload of the nursing staff. The development of protocols and records for the evaluation of results in daily practice in hospitals that use SC technology is recommended. Planning to manage changes to your workflow is an important precursor to implementation.	Breed et al.,^33^ Shaw et al.^39^
Organizational aspects focused on accessibility and feasibility	
Equal access of patients to SC is a concern for the group of professionals since the number of patients exceeds the availability of machines, which can lead to unequal access to care in a universal health system.	Shaw et al.^39^
The subsequent cooling time of the SC is added to the treatment time, so the chemotherapy session space is not available to another/patient. It is necessary to ensure that patients, regardless of whether or not they use SC technology, do not exceed waiting times for treatment.	Roe,^35^ Shaw et al.^39^
To address accessibility issues, it has been proposed: (a) to match the postcooling period with the administration of cytostatic agents that do not produce alopecia, when those are prescribed, (b) provide an additional room to which the patient can move with SC technology during the postcooling period and (c) assess using SC technology to enable two patients to be treated at once in open rooms.	Fischer‐Cartlidge et al.^42^
For successful implementation, the support of the organization is necessary. Organizational support includes both increased funding for nursing time and provision of additional space to accommodate increased treatment time, as well as SC therapy‐free spaces to reduce expectations of access for patients whom cannot receive this. Regarding the implementation of SC systems, it is necessary to consider the need for an interprofessional team, working with facility teams, training, taking into account medical resources and legal considerations, integrating technology into documentation, records and orders and good planning. The oncology nursing team could conduct patient education and reinforce adherence, which may positively affect their outcomes with SC technology.	Fischer‐Cartlidge et al.,^42^ Heery et al.^43^

Abbreviations: CIA, chemotherapy‐induced alopecia; SC, scalp cooling.

### 3.1 Ethical aspects focused on equal access, gender equity and doctor–patient communication supported by PtDAs

The main concern expressed by healthcare professionals was the patients' access to SC, as for many there can be a lack of equal access.^39^ On the one hand, this problem is mainly related to the limited availability of devices that affects accessibility and patient expectations.^39^ On the other hand, in the healthcare context, although a large percentage of professionals (85%) believe that both women and men need support to discuss their concerns about CIA,^40^ there seems to be a tendency for nurses to recommend SC more to women than to men.^40^ Thus, the availability of SC and adequate information provided to the patient to make a decision are two essential attributes to contributing to equal access and gender equity.^33^, ^35^

Communication between professionals and patients about how to cope with alopecia and available therapies to reduce it, such as SC, encourages evidence‐based informed and shared decision‐making. According to the nursing professionals' point of view, patients are not sufficiently informed about the associated risks of SC. Moreover, most nurses consider that patients are discouraged due to the required long duration of its use.^40^ Communicating SC characteristics on correct hair preservation, restoration, care and maintenance precautions (expected outcomes and appropriate, patient‐adjusted expectations) can reduce concerns and distress about CIA.^34^ However, according to individual characteristics, it is necessary to know the most appropriate timing of treatment to enhance a positive experience with SC.^35^, ^37^ A study highlighted that the nursing staff is one optimal profile to inform about SC to chemotherapy‐treated patients', as medical staff tend to offer SC to a lesser degree.^36^ The findings of a qualitative study conducted with patients treated with SC to reduce CIA indicate that information about the efficacy of SC provided by oncology staff was verbal and within the framework of professional experience.^38^ Patients reported that the information received on the SC process in terms of tolerability and hair care preservation, during treatment, was insufficient. However, patients with CIA who did not undergo SC reported that they did not receive information from their healthcare professional about SC as part of the treatment choice process. These patients were informed about SC through their peers, with no possibility of access to SC.

Finally, to facilitate effective communication about CIA and SC, and encourage informed decision‐making among practitioners and patients, some authors have promoted the development of a PtDA in web format.^41^ The PtDA is included in a website (https://www.scalpcooling.org/) which contains scientific information on alopecia and SC. Patients can complete a value clarification exercise here about their hair and how they perceive their personal likelihood of hair loss, as well as the safety of SC, ultimately receiving an overview with their own reasons for whether or not to choose the SC procedure, which can be printed out to discuss with healthcare professionals. When patients use PtDAs to make decisions, they gain more valid and accurate knowledge about the risks and benefits of available alternatives and are more likely to make decisions more consistent with their values.^41^

### 3.2 Patients' perspective and acceptability

The acceptability of SC among patients was high when applied in cases where this therapy has shown good results. In the pilot study by Dougherty,^37^ 50% of participants deemed that SC was worthwhile, and 50% also reported that they would use SC again. In another study, 82.22% of participants who obtained positive results with SC would recommend its use compared to 11.11% of those who obtained negative results.^28^

A factor affecting the acceptability of SC is the need for information before, during and after SC treatment.^41^ Other factors influencing the acceptability of SC are interpersonal speed differences in hair growth (to initiate or continue therapy with SC). Nursing staff attitudes towards SC were also considered a key factor for initiation and continuity with SC.^39^

In contrast, reported barriers to accepting SC were the nonguarantee that SC maintains hair; increased hospital stay; possible risk of skin metastases on the scalp; coldness of the technique; perception of pain when using SC; additional time requirement, preferring hair colouring to mask grey hair, finding using colour powder for touch‐ups too time‐consuming or finding washing hair only once a week intolerable.^31^, ^38^, ^41^, ^43^

From the patients' perspective, the majority of patients with breast cancer report that SC is poorly known before cancer diagnosis (73%).^31^ Regarding the information received, 63% of cancer patients are satisfied with the information provided by hospital staff about SC, while 56% of patients dissatisfied with the information reported not having received information about SC.^31^ Regarding comfort, the study of Massey^44^ revealed that 85% of participants considered SC comfortable.

An important aspect to consider is the patients' feelings after SC treatment are: improved self‐esteem (52%), improvement in quality of life (50%), disappointment (27%), hope (21%), insecurity (10%) and grief (6%).^31^ From a psychological point of view, uncertainty regarding hair preservation in patients using SC may cause further distress, and severe alopecia despite SC may lead to additional disappointment.^33^ In this sense, the qualitative study performed by Shaw et al.^38^ on women with breast cancer found that despite the explanation received about the possibility of hair loss even using SC, some women did not match prior expectations with SC experience and experienced considerable anguish when losing their hair. Another source of stress for women who used the SC was hair care, due to the lack of information about the products to use, the frequency with which to wash their hair or because they did not know what information they should give to their hairdressers. Other women reported less hair loss than expected and were satisfied with SC's contribution to maintaining a better quality of life during chemotherapy. Nevertheless, women who did not use SC experienced hair loss but, as this was an expected consequence of chemotherapy, they reported less shock and were more likely to monitor and plan for hair loss by shaving their heads once hair loss began. In this study, the level of pain and discomfort associated with SC was considered insufficient to discontinue treatment, although some women reported that discomfort was too great to repeat a future treatment.

While positive results with SC predict better body image, compared to people who have negative outcomes or to those who do not use SC,^30^, ^32^ women using SC have a greater fear of radical mastectomy, scalp metastasis and alopecia.^32^

### 3.3 Professional perspective and acceptability

The acceptability of SC for healthcare professionals seems to be moderate. Although for most (85%) oncology healthcare professionals, SC is known to prevent CIA; only 50% consider its implementation in the chemotherapy unit acceptable.^29^ On the one hand, the main facilitators for professionals' acceptability of SC are: seeing SC as a patient service^31^; responding to patient requests for access^39^; patient satisfaction with the experience^39^ and professionals' personal attitudes towards the need for alopecia intervention and personal attitudes towards SC.^39^ On the other hand, limited evidence on the efficacy and safety of SC mainly on the risk of scalp skin metastases, are the most important reasons for medical staff not supporting its use.^31^, ^39^, ^42^ In addition, logistic difficulties in the hospital are the main reasons precluding nursing staff from supporting SC.^31^ The nonavailability of SC in facilities on a routine basis, as well as the additional time and professional effort required by nursing staff, and limited access to SC were identified as the main barriers by healthcare professionals (not only nurses).^39^ The lack of knowledge about which patient profiles benefit most from SC,^39^ as well as difficulty identifying billing costs and processes without a similar intervention^42^ have also been pointed out as barriers. Regarding the lack of knowledge, Peerbooms et al.^31^ reported that 60% of nursing staff sought information about SC in scientific resources and exchanged knowledge with other professionals; 16% claimed the need for frequent SC training.^31^

Finally, Lemieux et al.^29^ reported that 88% of oncology healthcare staff believed that clinical trials on SC were necessary, and consequently, 85% would recommend their patients start a clinical trial on SC.

### 3.4 Organizational aspects focused on accessibility and feasibility

Feasibility seems to be one of the main problems from healthcare professionals' perspectives. Thus, 72% of healthcare professionals do not consider it feasible to extend patients' stay in the treatment room to receive SC after the chemotherapy session has finished.^29^ In terms of workload, limitations in trained nursing staff were considered an impediment to the routine use of SC.^39^ Lack of continuous healthcare professional training, as well as lack of knowledge about SC, generates worse results in patients, which fosters the view that it is not worth the extra time and extra nursing work.^39^

Regarding the implementation of SC, several studies have reported barriers and facilitators.^39^ Reported barriers include system‐level change in patient flow and working practices; lack of organizational support to increase funding for training and nursing staff time, and provision of additional space to see to increased time during chemotherapy treatment.^39^ From the professionals' point of view, these authors found that physician and nurse involvement in decision‐making was a relevant facilitator to implement SC. The study by Heery et al.^43^ considered that a key aspect of SC implementation is to ensure the availability of SC‐trained individuals to accompany patients eligible to participate in SC programmes. During the implementation of SC, the best‐trained people were female healthcare providers, patients or former caregivers. Ensuring patient adherence is also an essential component of SC implementation.

Some initiatives to improve the implementation of SC have been reported. According to Fischer‐Cartlidge et al.,^42^ some improvement initiatives are (1) matching the postcooling period with the administration of cytostatic agents that do not produce alopecia when those are prescribed; (2) enabling an additional room where the patient can move to with the device during the postcooling period and (3) assessing the possibility of using machines that enable two patients to be treated at the same time in open rooms. The need for real‐world data monitoring and evaluation is also relevant.^33^ Recording success rates with SC by involving patients in recording hair loss, together with resource use and costing data will add valuable information at health policy, managerial, clinical and patient decision levels. In this way, the outcomes assessment in daily clinical practice in some hospitals in The Netherlands proved to be very effective for chemotherapy programmes.^33^

## 4 DISCUSSION

The main goal of this SR was to assess the ethical, legal, organizational and social issues involved in SC use by oncologic patients. Overall, the quality of the studies included was low‐moderate. Findings were organized according to relevance into the following topics: (1) ethical aspects focused on equal access, gender equity and doctor–patient communication supported by PtDAs; (2) patient perspective and acceptability; (3) professional perspective and acceptability; (4) organizational aspects focused on accessibility and feasibility.

First, the review has pointed out the necessity to avoid the possibility of gender inequity when implementing the SC system. Thus, to avoid gender inequity, SC therapy should be offered equally to men and women, which avoids the assumption that men care less than women about maintaining their hair. In this regard, having clear protocols on which population to offer this therapy to and how to offer it can help minimize the influence of professionals' attitudes towards both CIA and SC.^31^, ^39^ However, the introduction of SC technology should not affect the ability to treat other patients, so waiting times for chemotherapy treatment should not be affected in any case.^35^, ^39^ This poses an organizational planning challenge, especially in properly managing the postcooling time that needs to be added to the chemotherapy regimen time. This is one of the key aspects that must be correctly planned before implementing the technology. Other ethical issues related to SC, such as whether SC should have some restrictions regarding age considerations, were not included in this review because no evidence was found addressing this problematic topic.

From the patients' perspective, the present review has shown how important it is to have enough detailed information before and during the use of SC, not only regarding the technology, but also hair care in general. When SC is available, adequate information and communication dynamics need to be implemented with all patients so that they can decide according to their values and preferences. To do this, decision support tools could be of great value,^41^ since these tools aid patients to clarify their own values about CIA and the impact of this on their own lives. The experience and feelings of each patient may be different, and for some of them, the prior expectations about SC might not be met by the results, which can lead to feelings of considerable anguish if they lose their hair. Due to the aforementioned, the nurses' and doctors' communication with patients and information provided about SC therapy is essential to reduce an imbalance between expectations and patient outcomes; as well as to identify potential sources of distress regarding hair preservation.^33^, ^37^, ^38^ Beyond providing information on the efficacy and safety of SC, information on hair care before, during and after the sessions is an important aspect for patients in chemotherapy treatment.^41^ Patients can complete a value clarification exercise about their hair and how they perceive their personal likelihood of hair loss, as well as the safety of SC, ultimately receiving an overview with their own reasons about whether or not to choose the SC procedure, which can be printed out for consultation with healthcare professionals. When patients use PtDAs to make decisions, they gain more valid and accurate knowledge about the risks and benefits of available alternatives and are more likely to make decisions more consistent with their values.^41^

From an organizational point of view, patient access to SC should be ensured in circumstances where the demand exceeds the availability of devices to minimize access inequalities.^39^ For this reason, the number of devices that each centre will require, the increase in nursing staff required, as well as the adequacy of additional space, must be carefully analysed and managed.^42^ The possibility of using machines that enable two patients to be treated at the same time in open rooms, as well as optimizing the use of SC machines is another factor that may help reduce the nursing workload.^42^

Regarding the implementation of SC, the literature points out some aspects worth highlighting. A crucial element for the successful implementation of this technology is planning ahead to manage changes in the workflow of the healthcare system. Healthcare managers should design an implementation plan with the help of the centre's interprofessional team, which could increase the acceptability of SC technology by professionals once it is implemented.^39^ Good planning should also include the recording systems to be introduced, which will enable the technology's impact to be assessed.^33^ Increased workload of the nursing staff can be reduced by strategies such as involving patients in the hair loss registry, preferably online.^33^

Moreover, Nangia^45^ stressed in a commentary that, due to the fact that the use of these devices requires new processes to be implemented by infusion centres, some providers and administrators may think it is too much effort to offer this service.

This SR provides results that are of interest to all the stakeholders involved in SC. Firstly, for patients who are interested in using SC, this SR can provide a bigger picture of the available evidence about patients' perspectives on this technology, which can be helpful to better inform their personal decisions. Second, doctors and nurses who may be interested in using SC in their centres could find these findings of great value. Third, the results of the present SR can also have an impact on policymaking when the healthcare systems are considering whether to introduce SC. In the case of SC, as the report^19^ shows, at the present time, it is not possible to recommend the use of SC for the prevention of CIA in women with breast cancer from the evidence available on effectiveness and cost‐effectiveness. This is mainly due to the low certainty associated with the long‐term safety of the technology. In these cases of uncertainty, it is even more relevant for policymakers to consider other factors involved in the technology, such as those considered in this SR. Considering all these factors can help to improve decision‐making from the policy‐making point of view. And finally, it also might be interesting for the industry to better understand the main concerns that healthcare professionals find regarding the feasibility of the technology.

### 4.1 Strengths and limitations of the review

This study has some strengths and offers valuable information for patients, healthcare professionals, managers and policymakers. Despite the recent publication of an SR on the efficacy and safety of SC,^13^ no specific publications have reported on patients' and professionals' perceptions and attitudes in terms of ethical, legal, organizational and social aspects. The SR by Rugo et al.^13^ reported on the effectiveness of SC and overlooked the attitudes and perceptions of patients and healthcare professionals, as well as the ethical, legal, organizational and social issues involved in the widespread use of SC. The present review is the first to address patients' and professionals' perspectives on SC therapy, as well as ethical, legal and organizational aspects. Another strength of the study is the ability to integrate all the actors involved in an evaluation process in a single search, from the organizational and institutional level to the patient's point of view, including the professionals' point of view.

However, this SR has certain limitations. First, the possibility that some studies have not been included because they are not drawn up in English or Spanish or because they are not indexed in the databases consulted. Another limitation is the type of study design; studies with a more robust methodology, which takes into account all patient aspects (from ethical to acceptability) are necessary.

This SR has tried to encompass all factors involved in the assessment of technology, from organizational to more person‐centred levels. Future SRs could focus on an analytical and critical review of this model, offering sustainable improvements in each step proposed. One study included in this review reported that, when patients use PtDA, in addition to helping them clarify their values and preferences, they had more realistic expectations about the possible benefits and side effects of their treatment options.^41^ Evidence has shown that when healthcare professionals receive training focused on a person‐centred care (PCC) model in the hospital setting, it improves their knowledge of the model and enables them to educate their patients in its use, with the help of PtDAs.^46^ Therefore, future research could focus on the evaluation of training programmes on the PCC model for healthcare professionals in this area, and on the development and assessment of PtDAs to make better‐informed decisions together with their practitioner.

## 5 CONCLUSIONS

This SR provides important learning points relevant to patient care, decision making and organizational policy. When ethical, legal, organizational and social issues related to the use of SC for the prevention of CIA were examined, the importance of equal access, which includes the need to offer SC to everybody, without assuming gender differences, to address concerns about hair loss and impaired perception of body self‐image was revealed. From a communication and educational perspective, the PtDa was found to be relevant to assist patients in clarifying their values and preferences, as well as the need for good communication with the healthcare staff team to adjust patients' prior expectations to reduce the potential distress associated with hair loss during SC use. This is important because the SR revealed that even when patients have received information, their expectations can differ and when patients use a PtDa they have more accurate expectations of the possible benefits and drawbacks of their options and are more likely to make decisions that are consistent with their values. In summary, we believe that this SR can benefit patient decisions, communication among healthcare staff and broader organizational considerations about SC. It can also help policymakers in deciding whether to include SC in their services, integrating other aspects complementary to efficacy and cost‐effectiveness. The integration of all relevant ethical, legal, organizational and social aspects can facilitate a more accurate and better‐informed decision‐making process.

## AUTHOR CONTRIBUTIONS

**Janet Delgado Rodríguez and Vanesa Ramos‐García**: Investigation; conceptualization; supervision and data analysis; writing – original draft; review and editing. **Diego Infante‐Ventura and María del Mar Trujillo‐Martín**: Methodology; writing – original draft, review and editing. **José Carlos Suarez‐Herrera and Antonio Rueda‐Domínguez**: Writing – review and editing. **Pedro Serrano‐Aguilar**: Writing – original draft, review and editing.

## CONFLICT OF INTEREST

J. D. R., V. R. G., D. I.‐V., P. S. A. and M. M. T. work for the same research entity that received the funding. The remaining authors declare no conflict of interest.

## Supporting information

Supplementary information.

Supplementary information.

Supplementary information.

## Data Availability

Data are available on request from the authors.
