# Supplementary material for: Ethical, legal, organizational and social issues related to the use of scalp cooling for the prevention of chemotherapy‐induced alopecia: A systematic review
Source: Health Expect. 2022 Dec 30;26(2):567–78. doi: 10.1111/hex.13679 (PMC10010082; doi:10.1111/hex.13679)
Supplement: Supplementary file 2 — Supplementary information. [file HEX-26--s003.docx]

**Search Strategy**

| **Medline**   \| 1 \| (chemotherapy-induced hair loss or chemotherapy-induced alopecia or chemotherapy-related alopecia or "CIA").ti,ab. \| 4795 \| \| --- \| --- \| --- \| \| 2 \| (chemotherapy adj5 (alopecia or hair loss or baldness)).ti,ab. \| 480 \| \| 3 \| (alopecia or hair loss or baldness).ti,ab. \| 21824 \| \| 4 \| 1 or 2 or 3 \| 26312 \| \| 5 \| (cooling scalp or scalp cooling or scalp-cooling or scalp hypothermia or cooling cap or hypothermic cap or scalp cyrogenic or cold cap or cooling system* or Hypothermia).ti,ab. \| 34777 \| \| 6 \| (dignilife or dignicap).ti,ab. \| 9 \| \| 7 \| (elastogel or Penguin Cold Caps).ti,ab. \| 1 \| \| 8 \| 5 or 6 or 7 \| 34777 \| \| 9 \| 4 and 8 \| 179 \| \| 10 \| ((("semi-structured" or semistructured or unstructured or informal or "in-depth" or indepth or "face-to-face" or structured or guide) adj3 (interview* or discussion* or questionnaire*)) or (focus group* or qualitative or ethnograph* or fieldwork or "field work" or "key informant")).ti,ab. or interviews as topic/ or focus groups/ or narration/ or qualitative research/ \| 426098 \| \| 11 \| exp Ethics/ or ethics.fs. or (ethic* or bioethic* or unintended consequences or (skills and training) or (benefits and harms) or beliefs or patient* autonomy or dignity or moral* or religio* or human rights or humanism or patient* integrity or principlism or normativ* or principle-base* or beneficence or non-maleficence or philosoph* or aristoteles or socrates or justice or fairness or patient* expectation or patient* accessibility or Beauchamp or childress or wide reflective* or socratic or social shaping or casuistry or coherence analy* or eclectic* or right to die or right to life or social value* or ethnic value* or personal value* or benefit-harm or harm-benefit or elsi or elsa).ab. /freq=2 \| 291129 \| \| 12 \| exp Privacy/ or exp Disclosure/ or exp Fraud/ or exp Government Regulation/ or exp Healthcare Disparities/ or exp Health Services Accessibility/ or exp Humanism/ or exp Human Rights/ or exp Insurance Coverage/ or exp Insurance, Health, Reimbursement/ or exp Jurisprudence/ or exp Legislation as Topic/ or Legislation & Jurisprudence.fs. or exp Patient Rights/ or exp Personhood/ or exp Prejudice/ or exp Professional Autonomy/ or exp Professional Misconduct/ or exp Social Stigma/ or exp Social Values/ or exp Stereotyping/ or exp Uncertainty/ or (((Healthcare or Health Care or nonclinical or Community Based) adj (Deliver* or Distribution* or System*)) or (legal* or liabilit* or litigation* or constitutional or justice or law or laws or jurisprudence or complicit* or privacy or private or confidential*) or ((care or treatment) adj2 (duty or obligat*)) or (social* adj (responsibl* or obligat*)) or ((informed or presumed) adj2 (choice or decision making))).ti,ab,kf. or (basic right* or access* right or duty to know or equally access* or external pressure or fundamental right* or human right* or informed choice or informed decision making or public pressure or regulatory frame* or right of access* or right to know or acceptance or accessibility or accountability or autonomy or beneficence or civil right* or communitarian* or community values or confidentiality or coverage or dignity or directive or disclosure or discrimination or elsi or elsis or equality or equity or fairness or freedom or harm or inequalit* or inequity or patient* integrity or justice or law or lawmaker* or lawsuit* or lawyer* or legacy or legal* or legislation or legitimacy or liability or litigation or medicolegal or non-coverage or nonmaleficence or non-maleficence or normative or normativity or permissibility or prejudice* or privacy or reimburse* or social values or stigma or stigmas or stigmatisation or stigmatise or stigmatization or stigmatize or transparency or uncertainty or value judgment).ab. /freq=2 \| 1245503 \| \| 13 \| Decision Making, Organizational/ or Efficiency, Organizational/ or Models, Organizational/ or Organizational Objectives/ or exp Personnel Management/ or exp Professional Practice/ or "Organization and Administration"/ or Healthcare Common Procedure Coding System/ or Case Managers/ or Program Development/ or "Organization and Administration".fs. or organizational.ti,ab,kw. or ((organization* or work or workflow or work flow or work planning or implementation* or information needs or need assessment* or skills or attitud* or culture or patient path or user path or client path or quality assurance or sustainability or centralization or decentralization or hospital management or manager* or supplier*) adj9 (patient* or client* or user or users or practice* or hospital* or home* or primary care or clinical or Medical or nurse* or physician* or profesional*)).ti,ab. \| 870061 \| \|  \|  \|  \| \| 14 \| ((social or burden* or Impact* or choice* or information needs or communication* or self-care or self-management or trade-off) adj5 (social or patient*)).ti. \| 158805 \| \| 15 \| exp patient acceptance of health care/ or caregivers/ or exp Patient Preference/ or exp Patient Satisfaction/ or ((Patient* or individual* or person* or care-giver* or caregiver* or client or communit* or consumer* or public* or user* or sufferer* or suffering or diseased or troubled or sick* or invalid or people or patient* or individual* or person* or carer or caregiver* or client or communit* or consumer* or public* or user* or sufferer* or suffering or diseased or troubled or sick* or people or patient or patients or proband* or individuals or survivor* or family or families or familial or kindred* or relative or relatives or care giver* or caregiver* or carer or carers or personal or spous* or partner or partners or couples or users or participant* or people or child* or teenager* or adolescent* or youth or girls or boys or adults or elderly or females or males or women* or men or men's or mother* or father* or parents or parent or parental or maternal or paternal) adj5 (prescrib* or Elicit* or Choice* or Logit or Adheren* or Preferen* or service-user or preference* or opinion* or perception* or perspective* or view* or voice* or experienc* or expectation* or wish or wishes or attitud* or lifespan* or refus* or patient autonomy or activities of daily living or quality of life or everyday life)).ti,kw. \| 455762 \| \| 16 \| ((patient* or individual* or person* or carer or caregiver* or client or communit* or consumer* or public* or user* or sufferer* or suffering or diseased or troubled or sick* or people or patient or patients or proband* or individuals or survivor* or family or families or familial or kindred* or relative or relatives or care giver* or caregiver* or carer or carers or personal or spous* or partner or partners or couples or users or participant* or people or child* or teenager* or adolescent* or youth or girls or boys or adults or elderly or females or males or women* or men or men's or mother* or father* or parents or parent or parental or maternal or paternal) adj2 (service-user or preference* or opinion or perception* or perspective* or view* or voice* or experience* or expectation* or wish or wishes or attitud* or Wellbeing or well-being or self-care or self* or belief* or concern* or worr* or burden* or problem* or distress or psychology* or social activit* or famil* or friend* or emotion* or satisf* or dissatisf* or happ* or unhapp* or behav* or lifestyle or routine or life or autonomy or activities of daily living or quality of life or everyday life or skeptic* or enthusias* or daily lives or frequent-or-daily-users or Acceptanc*)).ti,kw. \| 435950 \| \| 17 \| 15 or 16 \| 753114 \| \| 18 \| exp *pollution/ or exp *pollutant/ or *environmental exposure/ or exp *environmental impact/ or *ecotoxicology/ or *exp biota/ or exp *environmental health/ \| 845130 \| \| 19 \| (waste* or pollution* or polluting or pollutant* or contamination* or contaminated or environmental health).ti,kw. \| 147141 \| \| 20 \| ((hazardous or toxic or toxicity or toxin or toxins or risk or risks or impact or impacts) adj5 environment*).ti,ab,kw. \| 73038 \| \| 21 \| (natural environment* or soil or soils or flora or floras or fauna or faunas or renewable resource*).ti,kw. \| 83020 \| \| 22 \| (waste* or contamination*).ti,kf. \| 92562 \| \| 23 \| (pollution* or polluting or pollutant* or contaminated or environmental health).ti,ab,kf. \| 236873 \| \| 24 \| (natural environment* or soil or soils or flora or floras or fauna or faunas or renewable resource*).ti,ab,kf. \| 224784 \| \| 25 \| (environment* adj5 sustainabilit*).ti,ab,kf. \| 2837 \| \| 26 \| carbon footprint*.ti,ab,kf. \| 1143 \| \| 27 \| (environment* adj2 (assess* or impact* or outcome* or implication* or consideration*)).ti,ab,kf. \| 30572 \| \| 28 \| environment*.ti,kf. \| 202106 \| \| 29 \| environmental*.ab. \| 492608 \| \| 30 \| ((health technology assessment or HTA or HTAs) and environmental*).ti,ab,kf. \| 62 \| \| 31 \| ((health technology assessment or HTA or HTAs) adj7 environment).ti,ab,kf. \| 25 \| \| 32 \| 18 or 19 or 20 or 21 or 22 or 23 or 24 or 25 or 26 or 27 or 28 or 29 or 30 or 31 \| 1556534 \| \| 33 \| 9 and 10 \| 4 \| \| 34 \| 11 or 12 or 13 or 14 or 17 \| 2744177 \| \| 35 \| 9 and 34 \| 26 \| \| 36 \| 9 and 32 \| 5 \| |
| --- | --- | --- | --- | --- | --- | --- | --- | --- | --- | --- | --- | --- | --- | --- | --- | --- | --- | --- | --- | --- | --- | --- | --- | --- | --- | --- | --- | --- | --- | --- | --- | --- | --- | --- | --- | --- | --- | --- | --- | --- | --- | --- | --- | --- | --- | --- | --- | --- | --- | --- | --- | --- | --- | --- | --- | --- | --- | --- | --- | --- | --- | --- | --- | --- | --- | --- | --- | --- | --- | --- | --- | --- | --- | --- | --- | --- | --- | --- | --- | --- | --- | --- | --- | --- | --- | --- | --- | --- | --- | --- | --- | --- | --- | --- | --- | --- | --- | --- | --- | --- | --- | --- | --- | --- | --- | --- | --- | --- | --- | --- | --- |

| **EMBASE**   \| 1 \| 'chemotherapy-induced hair loss':ti,ab OR 'chemotherapy-induced alopecia':ti,ab OR 'chemotherapy-related alopecia':ti,ab OR 'cia':ti,ab \| 7,352 \| \| --- \| --- \| --- \| \| 2 \| (chemotherapy NEAR/5 (alopecia OR 'hair loss' OR baldness)):ti,ab \| 810 \| \| 3 \| alopecia:ti,ab OR 'hair loss':ti,ab OR baldness:ti,ab \| 32,161 \| \| 4 \| #1 OR #2 OR #3 \| 38,977 \| \| 5 \| cooling scalp':ti,ab OR 'scalp cooling':ti,ab OR 'scalp-cooling':ti,ab OR 'scalp hypothermia':ti,ab OR 'cooling cap':ti,ab OR 'hypothermic cap':ti,ab OR 'scalp cyrogenic':ti,ab OR 'cold cap':ti,ab OR 'cooling system*':ti,ab OR hypothermia:ti,ab \| 45,431 \| \| 6 \| dignilife:ti,ab OR dignicap:ti,ab \| 43 \| \| 7 \| elastogel:ti,ab OR 'penguin cold caps':ti,ab \| 5 \| \| 8 \| #5 OR #6 OR #7 \| 45,432 \| \| 9 \| #4 AND #8 \| 366 \| \| 10 \| (((('semi structured' OR semistructured OR unstructured OR informal OR 'in-depth' OR indepth OR 'face-to-face' OR structured OR guide) NEAR/3 (interview* OR discussion* OR questionnaire*)):ti,de) OR (focus:ti,de AND group*:ti,de) OR qualitative:ti,de OR ethnograph*:ti,de OR fieldwork:ti,de OR 'field work':ti,de OR 'key informant':ti,de OR 'qualitative research'/exp) AND ([english]/lim OR [spanish]/lim) AND ('article'/it OR 'letter'/it OR 'note'/it OR 'review'/it OR 'article in press'/it) \| 149,49 \| \| 11 \| 'ethics'/exp OR 'ethics' OR ethic*:ti,de OR bioethic*:ti,de OR 'unintended consequences':ti,de OR 'skills and training':ti,de OR 'benefits and harms':ti,de OR beliefs:ti,de OR 'patient* autonomy':ti,de OR dignity:ti,de OR moral*:ti,de OR religio*:ti,de OR 'human rights':ti,de OR humanism:ti,de OR 'patient* integrity':ti,de OR principlism:ti,de OR normativ*:ti,de OR 'principle base*':ti,de OR beneficence:ti,de OR 'non maleficence':ti,de OR philosoph*:ti,de OR aristoteles:ti,de OR socrates:ti,de OR justice:ti,de OR fairness:ti,de OR 'patient* expectation*':ti,de OR 'patient* accessibility':ti,de OR beauchamp:ti,de OR childress:ti,de OR 'wide reflective*':ti,de OR socratic:ti,de OR 'social shaping':ti,de OR casuistry:ti,de OR 'coherence analy*':ti,de OR eclectic*:ti,de OR 'right to die':ti,de OR 'right to life':ti,de OR 'social value*':ti,de OR 'ethnic value*':ti,de OR 'personal value*':ti,de OR 'benefit harm':ti,de OR 'harm benefit':ti,de OR elsi:ti,de OR elsa:ti,de \| 497,918 \| \| 12 \| 'legal aspect'/mj OR 'law'/exp OR 'justice'/exp OR 'jurisprudence'/mj OR 'patient right'/exp OR 'personhood'/de OR 'prejudice'/exp OR 'privacy'/exp OR 'fraud'/exp OR 'government'/exp OR 'health care disparity'/mj OR 'health services accessibility'/mj OR 'humanism'/mj OR 'human rights'/mj OR 'reimbursement'/mj OR (((healthcare OR 'health care' OR nonclinical OR 'community based') NEAR/1 (deliver* OR distribution*)):ti,ab) OR legal*:ti,de OR liabilit*:ti OR litigation*:ti,ab,de OR constitutional:ti,ab,de OR justice*:ti,de OR law:ti,de OR laws:ti,de OR jurisprudence*:ti,de OR complicit*:ti,ab,de OR privacy:ti,ab,de OR confidential*:ti,ab,de OR (((care OR treatment) NEAR/2 (duty OR obligat*)):ti,ab,de) OR ((social* NEAR/1 (responsibl* OR obligat*)):ti,ab,de) OR (((informed OR presumed) NEAR/2 (choice OR 'decision making')):ti,ab,de) OR 'basic right*':ti OR 'duty to know':ti OR 'equally access*':ti OR 'external pressure*':ti OR 'fundamental right*':ti OR 'human right*':ti OR 'informed choice*':ti OR 'informed decision*':ti OR 'public pressure*':ti OR 'regulatory frame*':ti OR 'right of access*':ti OR 'right to know':ti OR acceptance:ti OR accessibility:ti OR accountability:ti OR autonomy:ti OR beneficence:ti OR 'civil right*':ti OR communitarian*:ti OR 'community value*':ti OR confidentiality:ti OR dignity:ti OR directive:ti OR disclosure:ti OR discrimination:ti OR equality:ti OR equity:ti OR fairness:ti OR freedom:ti OR harm:ti OR inequalit*:ti OR inequity:ti OR 'patient* integrity':ti OR justice:ti OR law:ti OR lawmaker*:ti OR lawsuit*:ti OR lawyer*:ti OR legacy:ti OR legal*:ti OR legislation:ti OR legitimacy:ti OR liability:ti OR litigation:ti OR medicolegal:ti OR 'non coverage':ti OR nonmaleficence:ti OR 'non maleficence':ti OR normative*:ti OR normativity:ti OR permissibility:ti OR prejudice*:ti OR privacy:ti OR reimburse*:ti OR transparency:ti OR uncertainty:ti OR 'value judgment':ti \| 855,195 \| \| 13 \| 'organization'/mj OR 'personnel management'/mj OR 'professional practice'/mj OR 'organization and management'/mj OR 'healthcare common procedure coding system'/mj OR 'case manager'/mj OR 'program development'/mj OR organizational:ti,de OR 'patient path':ti,ab,de OR 'user path':ti,ab,de OR 'client path':ti,ab,de OR (((organization* OR workflow OR 'work flow' OR 'work planning' OR implementation* OR 'information needs' OR 'need assessment*' OR skills OR attitud* OR culture OR 'quality assurance' OR sustainability OR centralization OR decentralization OR 'hospital managesocialment' OR manager* OR supplier) NEAR/9 (patient* OR client* OR user OR users OR practice* OR hospital* OR home* OR 'primary care' OR clinical OR medical OR nurse* OR physician* OR profesional* OR clinician*)):ti,ab) \| 496,505 \| \| 14 \| 'social acceptance'/mj OR 'social aspect'/mj OR 'social norm'/mj OR 'social problem'/mj OR 'social rejection'/mj OR 'social status'/mj OR 'social structure'/mj OR 'social aspects and related phenomena'/mj OR social:ti OR 'social factor':ti,de OR 'social aspect*':ti OR 'social norm':ti OR 'social burden*':ti OR 'social impact*':ti OR (((choice* OR 'information needs' OR communication* OR 'self care' OR 'self management' OR 'trade off' OR planning) NEAR/2 (social OR patient*)):ab) OR (((stigma* OR burden* OR impact* OR choice* OR 'information need*' OR communication* OR 'self care' OR selcare OR 'self management' OR 'trade off') NEAR/5 (patient* OR individual* OR person* OR 'care giver*' OR caregiver* OR carer OR carers* OR client OR communit* OR consumer* OR public* OR user* OR sufferer* OR suffering OR diseased OR troubled OR sick* OR invalid OR people OR proband* OR survivor* OR famil* OR familial OR kindred* OR relative* OR spous* OR partner* OR couple* OR participant* OR child* OR teenager* OR adolescent* OR youth OR girl* OR boy* OR adult* OR elder* OR older* OR female* OR male* OR women* OR men* OR mother* OR father* OR parent* OR matern*)):ti) \| 260,463 \| \| 15 \| 'patient attitude'/exp OR 'patient participation'/exp OR 'patient decision making'/exp OR 'patient preference'/exp OR 'patient satisfaction'/exp OR 'patient centered':ti,ab,de OR patientcentered:ti,ab,de OR 'patient center*':ti,ab,de OR patientcenter*:ti,ab,de OR 'profesional patient*':ti,ab,de OR (((patient* OR individual* OR person* OR 'care giver*' OR caregiver* OR 'care giver*' OR carer OR carers* OR client OR communit* OR consumer* OR public* OR user* OR sufferer* OR suffering OR diseased OR troubled OR sick* OR invalid OR people OR proband* OR survivor* OR famil* OR familial OR kindred* OR relative* OR spous* OR partner* OR couple* OR participant* OR child* OR teenager* OR adolescent* OR youth OR girl* OR boy* OR adult* OR elder* OR older* OR female* OR male* OR women* OR men* OR mother* OR father* OR parent* OR matern*) NEAR/1 (prescrib* OR elicit* OR choice* OR logit OR adheren* OR preferen* OR 'service user' OR preference* OR opinion* OR perception* OR perce* OR perspective* OR view* OR voice* OR experienc* OR expectat* OR wish OR wishes OR attitud* OR lifespan* OR refus* OR 'patient autonomy' OR 'activities of daily living' OR 'quality of life' OR 'everyday life' OR decisi* OR paticipat* OR involvement* OR desir* OR activation OR empower* OR adaptat* OR educat* OR knowledge*)):ti) OR (((patient* OR individual* OR person* OR 'care giver*' OR caregiver* OR 'care giver*' OR carer OR carers* OR client OR communit* OR consumer* OR public* OR user* OR sufferer* OR suffering OR diseased OR troubled OR sick* OR invalid OR people OR proband* OR survivor* OR famil* OR familial OR kindred* OR relative* OR spous* OR partner* OR couple* OR participant* OR child* OR teenager* OR adolescent* OR youth OR girl* OR boy* OR adult* OR elder* OR older* OR female* OR male* OR women* OR men* OR mother* OR father* OR parent* OR matern*) NEAR/1 (acceptanc* OR acceptab* OR valuation* OR wellbeing OR 'well being' OR 'self care' OR self* OR belief* OR concern* OR worr* OR burden* OR problem* OR distress OR psychology* OR 'social activit*' OR friend* OR emotion* OR satisf* OR dissatisf* OR happ* OR unhapp* OR behav* OR lifestyle OR routine* OR life* OR autonom* OR skeptic* OR enthusias* OR 'daily lives' OR daily OR 'frequent or daily users')):ti) \| 569,519 \| \| 16 \| 'pollution'/exp \| 400,403 \| \| 17 \| 'pollutant'/exp \| 336,196 \| \| 18 \| 'environmental exposure'/exp \| 114,371 \| \| 19 \| 'environmental impact'/exp \| 79,451 \| \| 20 \| 'ecotoxicology'/exp \| 8,697 \| \| 21 \| 'biota'/exp \| 162,614 \| \| 22 \| 'environmental health'/exp \| 40,64 \| \| 23 \| waste*:ti,kw OR pollution*:ti,kw OR polluting:ti,kw OR pollutant*:ti,kw OR contamination*:ti,kw OR contaminated:ti,kw OR 'environmental health':ti,kw \| 237,031 \| \| 24 \| ((hazardous OR toxic OR toxicity OR toxin OR toxins OR risk OR risks OR impact OR impacts) NEAR/5 environment*):ti,ab,kw \| 91,281 \| \| 25 \| 'natural environment*':ti,kw OR soil:ti,kw OR soils:ti,kw OR flora:ti,kw OR floras:ti,kw OR fauna:ti,kw OR faunas:ti,kw OR 'renewable resource*':ti,kw \| 107,097 \| \| 26 \| waste*:ti,de OR contamination*:ti,de \| 356,101 \| \| 27 \| pollution*:ti,ab,de OR polluting:ti,ab,de OR pollutant*:ti,ab,de OR contaminated:ti,ab,de OR 'environmental health':ti,ab,de \| 477,8 \| \| 28 \| (environment* NEAR/5 sustainabilit*):ti,ab,de \| 7,153 \| \| 29 \| 'carbon footprint*':ti,ab,de \| 9,007 \| \| 30 \| (environment* NEAR/2 (assess* OR impact* OR outcome* OR implication* OR consideration*)):ti,ab,de \| 69,395 \| \| 31 \| environment*:ti,de \| 772,163 \| \| 32 \| environmental*:ab \| 581,579 \| \| 33 \| ('health technology assessment':ti,ab,de OR hta:ti,ab,de OR htas:ti,ab,de) AND environmental*:ti,ab,de \| 93 \| \| 34 \| (('health technology assessment' OR hta OR htas) NEAR/7 environment):ti,ab,de \| 45 \| \| 35 \| #16 OR #17 OR #18 OR #19 OR #20 OR #21 OR #22 OR #23 OR #24 OR #25 OR #26 OR #27 OR #28 OR #29 OR #30 OR #31 OR #32 OR #33 OR #34 \| 1,903,843 \| \| 36 \| #9 AND #10 \| 3 \| \| 37 \| #11 OR #12 OR #13 OR #14 OR #15 \| 2,445,844 \| \| 38 \| #9 AND #37 \| 70 \| \| 39 \| #9 AND #35 \| 3 \| |
| --- | --- | --- | --- | --- | --- | --- | --- | --- | --- | --- | --- | --- | --- | --- | --- | --- | --- | --- | --- | --- | --- | --- | --- | --- | --- | --- | --- | --- | --- | --- | --- | --- | --- | --- | --- | --- | --- | --- | --- | --- | --- | --- | --- | --- | --- | --- | --- | --- | --- | --- | --- | --- | --- | --- | --- | --- | --- | --- | --- | --- | --- | --- | --- | --- | --- | --- | --- | --- | --- | --- | --- | --- | --- | --- | --- | --- | --- | --- | --- | --- | --- | --- | --- | --- | --- | --- | --- | --- | --- | --- | --- | --- | --- | --- | --- | --- | --- | --- | --- | --- | --- | --- | --- | --- | --- | --- | --- | --- | --- | --- | --- | --- | --- | --- | --- | --- | --- |

| **WOS**   \| 1 \| TI= ("chemotherapy-induced hair loss" or "chemotherapy-induced alopecia" or "chemotherapy-related alopecia" or "CIA") or AB= ("chemotherapy-induced hair loss" or "chemotherapy-induced alopecia" or "chemotherapy-related alopecia" or "CIA") ) \| 12.677 \| \| --- \| --- \| --- \| \|  \| TI= ("chemotherapy-induced hair loss" or "chemotherapy-induced alopecia" or "chemotherapy-related alopecia" or "CIA") or AB= ("chemotherapy-induced hair loss" or "chemotherapy-induced alopecia" or "chemotherapy-related alopecia" or "CIA") ) \|  \| \| 2 \| TS= (chemotherapy NEAR/5 (alopecia or "hair loss" or baldness) ) \| 1.129 \| \|  \|  \|  \| \| 3 \| TS= (alopecia or "hair loss" or baldness) \| 44.344 \| \| 4 \| #3 OR #2 OR #1 \| 56.436 \| \| 5 \| (TI= ("cooling scalp" or "scalp cooling" or "scalp-cooling" or "scalp hypothermia" or "cooling cap" or "hypothermic cap" or "scalp cyrogenic" or "cold cap" or "cooling system*" or Hypothermia) or AB= ("cooling scalp" or "scalp cooling" or "scalp-cooling" or "scalp hypothermia" or "cooling cap" or "hypothermic cap" or "scalp cyrogenic" or "cold cap" or "cooling system*" or Hypothermia) ) \| 98.588 \| \|  \| (TI= ("cooling scalp" or "scalp cooling" or "scalp-cooling" or "scalp hypothermia" or "cooling cap" or "hypothermic cap" or "scalp cyrogenic" or "cold cap" or "cooling system*" or Hypothermia) or AB= ("cooling scalp" or "scalp cooling" or "scalp-cooling" or "scalp hypothermia" or "cooling cap" or "hypothermic cap" or "scalp cyrogenic" or "cold cap" or "cooling system*" or Hypothermia) ) \|  \| \| 6 \| TS= (dignilife or dignicap) \| 27 \| \| 7 \| TS= (elastogel or "Penguin Cold Caps") \| 2 \| \| 8 \| #7 OR #6 OR #5 \| 98.596 \| \| 9 \| #8 AND #4 \| 369 \| \| 10 \| TS=(qualitative  OR ethnol*  OR ethnog*  OR ethnonurs*  OR emic  OR etic  OR leininger  OR noblit  OR "field note*"  OR "field record*"  OR fieldnote*  OR "field stud*" or "participant observ*"  OR "participant observation*"  OR hermaneutic*  OR phenomenolog*  OR "lived experience*"  OR heidegger*  OR husserl*  OR "merleau-pont*"  OR colaizzi  OR giorgi  OR ricoeur  OR spiegelberg  OR "van kaam"  OR "van manen"  OR "grounded theory"  OR "constant compar*"  OR "theoretical sampl*"  OR glaser  AND strauss  OR "content analy*"  OR "thematic analy*"  OR narrative*  OR "unstructured categor*"  OR "structured categor*"  OR "unstructured interview*"  OR "semi-structured interview*"  OR "maximum variation*"  OR snowball  OR audio*  OR tape*  OR video*  OR metasynthes*  OR "meta-synthes*"  OR metasummar*  OR "meta-summar*"  OR metastud*  OR "meta-stud*"  OR "meta-ethnograph*"  OR metaethnog*  OR "meta-narrative*"  OR metanarrat*  OR " meta-interpretation*"  OR metainterpret*  OR "qualitative meta-analy*"  OR "qualitative metaanaly*"  OR "qualitative metanaly*"  OR "purposive sampl*"  OR "action research"  OR "focus group*" or photovoice or "photo voice" or "mixed method*") \| 3.671.577 \| \|  \|  \|  \| \| 11 \| TS=(ethic*  OR bioethic*  OR "unintended consequences"  OR "skills and training"  OR "benefits and harms"  OR beliefs  OR "patient* autonomy"  OR dignity  OR moral*  OR religio*  OR "human rights"  OR humanism  OR "patient* integrity"  OR principlism  OR normativ*  OR "principle base*"  OR beneficence  OR "non maleficence"  OR philosoph*  OR aristoteles  OR socrates  OR justice  OR fairness  OR "patient* expectation*"  OR "patient* accessibility*"  OR beauchamp  OR childress  OR "wide reflective*"  OR socratic  OR "social shaping"  OR casuistry  OR "coherence analy*"  OR eclectic*  OR "right to die"  OR "right to life"  OR "social value*"  OR "ethnic value*"  OR "personal value*"  OR "benefit harm"  OR "harm benefit"  OR elsi  OR elsa) \| 2.267.160 \| \|  \|  \|  \| \| 12 \| TS=(privacy  OR fraud  OR "government regulation"  OR "health care disparity"  OR "health care delivery"  OR "humanism"  OR "human rights"  OR "insurance"  OR "reimbursement"  OR "jurisprudence"  OR law  OR "legislation and jurisprudence"  OR "patient right"  OR "prejudice"  OR "professional autonomy"  OR "professional misconduct")  OR TS=((healthcare  OR "health care"  OR nonclinical  OR "community based") NEAR/1 (deliver*  OR distribution*  OR system*) )  OR TS=(liabilit*  OR litigation*  OR constitutional  OR laws  OR jurisprudence  OR complicit*  OR private  OR confidential*)  OR TS=((care  OR treatment) NEAR/2 (duty  OR obligat*) )  OR TS=(social* NEAR/1 (responsibl*  OR obligat*) )  OR TS=((informed  OR presumed) NEAR/2 (choice  OR "decision making") )  OR TS=("basic right*"  OR "access* right"  OR "duty to know"  OR "equally access*"  OR "external pressure"  OR "fundamental right*"  OR "human right*"  OR "informed choice"  OR "informed decision making"  OR "public pressure"  OR "regulatory frame*"  OR "right of access*"  OR "right to know"  OR acceptance  OR accessibility  OR accountability  OR autonomy  OR beneficence  OR "civil right*"  OR communitarian*  OR "community values"  OR confidentiality  OR dignity  OR directive  OR disclosure  OR discrimination  OR elsi  OR elsis  OR equality  OR equity  OR fairness  OR freedom  OR harm  OR inequalit*  OR inequity  OR "patient* integrity"  OR justice  OR law  OR lawmaker*  OR lawsuit*  OR lawyer*  OR legacy  OR legal*  OR legislation  OR legitimacy  OR liability  OR litigation  OR medicolegal  OR "non coverage"  OR nonmaleficence  OR "non maleficence"  OR normative  OR normativity  OR permissibility  OR prejudice*  OR privacy  OR reimburse*  OR "social values"  OR stigma  OR stigmas  OR stigmatisation  OR stigmatise  OR stigmatization  OR stigmatize  OR transparency  OR uncertainty  OR "value judgment") \| 5.827.757 \| \|  \|  \|  \| \| 13 \| TI=(organization*)  OR TS=(("personnel management"  OR "professional practice"  OR "organization and management"  OR "healthcare common procedure coding system"  OR "case manager"  OR "program development"  OR "work planning"  OR implementation*  OR "information needs"  OR "need assessment*"  OR skills  OR attitud*  OR culture  OR "quality assurance"  OR sustainability  OR centralization  OR decentralization  OR "hospital management"  OR supplier*)  AND (patient*  OR client*  OR user  OR users  OR practice*  OR hospital*  OR home*  OR "primary care"  OR clinical  OR medical  OR nurse*  OR physician*  OR profesional*)) \| 2.933.877 \| \|  \|  \|  \| \| 14 \| TI=(social  OR "social burden*"  OR "social impact*"  OR "information needs"  OR "self care"  OR "self management"  OR "trade off") \| 6.685.82 \| \|  \|  \|  \| \| 15 \| TS=("care and caring"  OR "social acceptance"  OR "social aspect"  OR "social norm"  OR "social problem"  OR "social rejection"  OR "social status"  OR "social structure"  OR "social aspects and related phenomena"  OR "social burden*"  OR "social impact*"  OR choice*  OR "information needs"  OR communication*  OR "self care"  OR "self management"  OR "trade off") \| 3.996.133 \| \| 16 \| TS=("patient attitude"  OR "patient participation"  OR "patient decision making"  OR "patient preference"  OR "patient satisfaction")  OR TI=("service user"  OR preference*  OR opinion  OR perception*  OR perspective*  OR view*  OR voice*  OR deerience*  OR deectation*  OR wish  OR wishes  OR attitud*  OR wellbeing  OR "well being"  OR "self care"  OR self*  OR belief*  OR concern*  OR worr*  OR burden*  OR problem*  OR distress  OR psychology*  OR "social activit*"  OR famil*  OR friend*  OR emotion*  OR satisf*  OR dissatisf*  OR happ*  OR unhapp*  OR behav*  OR lifestyle  OR routine  OR life  OR autonomy  OR "activities of daily living"  OR "quality of life"  OR "everyday life"  OR skeptic*  OR enthusias*  OR "daily lives"  OR "frequent or daily users"  OR acceptanc*) \| 5.973.747 \| \| 17 \| #16 OR #15 OR #14 OR #13 OR #12 OR #11 \| 17.676.346 \| \| 18 \| (TS= (waste* or pollution* or polluting or pollutant* or contamination* or contaminated or environmental health)   ) \| 3.143.693 \| \| 19 \| (TS= ((hazardous or toxic or toxicity or toxin or toxins or risk or risks or impact or impacts)   NEAR/5 environment*)) \| 298.341 \| \| 20 \| (TS= (natural environment* or soil or soils or flora or floras or fauna or faunas or renewable resource*)   ) \| 2.716.522 \| \| 21 \| (TS= (environment* NEAR/5 sustainabilit*)   ) \| 28.159 \| \| 22 \| (TS= (environment* NEAR/2 (assess* or impact* or outcome* or implication* or consideration*)   )) \| 225.159 \| \| 23 \| (TS= carbon footprint*) \| 15.444 \| \| 24 \| (TS= ((health technology assessment)   NEAR/4 environment)) \| 7 \| \| 25 \| (TS= (HTA* NEAR/7 environment)   ) \| 44 \| \| 26 \| (TS= (pollution* or polluting or pollutant* or contaminated or environmental health)   ) \| 1.934.921 \| \| 27 \| #26 OR #25 OR #24 OR #23 OR #22 OR #21 OR #20 OR #19 OR #18 \| 5.064.891 \| \| 28 \| #10 AND #9 \| 7 \| \| 29 \| #17 AND #9 \| 61 \| \| 30 \| #27 AND #9 \| 2 \| |
| --- | --- | --- | --- | --- | --- | --- | --- | --- | --- | --- | --- | --- | --- | --- | --- | --- | --- | --- | --- | --- | --- | --- | --- | --- | --- | --- | --- | --- | --- | --- | --- | --- | --- | --- | --- | --- | --- | --- | --- | --- | --- | --- | --- | --- | --- | --- | --- | --- | --- | --- | --- | --- | --- | --- | --- | --- | --- | --- | --- | --- | --- | --- | --- | --- | --- | --- | --- | --- | --- | --- | --- | --- | --- | --- | --- | --- | --- | --- | --- | --- | --- | --- | --- | --- | --- | --- | --- | --- | --- | --- | --- | --- | --- | --- | --- | --- | --- | --- | --- | --- | --- | --- | --- | --- | --- | --- | --- | --- | --- | --- | --- | --- | --- | --- |
