# Supplementary material for: Ethical, legal, organizational and social issues related to the use of scalp cooling for the prevention of chemotherapy‐induced alopecia: A systematic review
Source: Health Expect. 2022 Dec 30;26(2):567–78. doi: 10.1111/hex.13679 (PMC10010082; doi:10.1111/hex.13679)
Supplement: Supplementary file 3 — Supplementary information. [file HEX-26--s002.docx]

| **Table 1. Assessment of methodological limitations with the instrument SANRA** | | | | | |
| --- | --- | --- | --- | --- | --- |
| **Questions** | | **Breed 2011** | **Roe 2014** | **Peterson 2020** | **Young 2016** |
| Justification of the article’s importance for the readership | | 2 | 2 | 1 | 1 |
| The importance is not justified | 0 |  |  |  |  |
| The importance is alluded to, but not explicitly justified | 1 |  |  |  |  |
| The importance is explicitly justified | 2 |  |  |  |  |
| Statement of concrete aims or formulations of questions | | 1 | 1 | 1 | 0 |
| No aims or questions are formulated | 0 |  |  |  |  |
| Aims are formulated generally but not concretely or in terms of clear questions | 1 |  |  |  |  |
| One or more concrete aims or questions are formulated 2 | 2 |  |  |  |  |
| Description of the literature search | | 0 | 0 | 0 | 0 |
| The search strategy is not presented | 0 |  |  |  |  |
| The literature search is described briefly | 1 |  |  |  |  |
| The literature search is described in detail, including search terms and inclusion criteria | 2 |  |  |  |  |
| Referencing | | 2 | 2 | 2 | 2 |
| Key statements are not supported by references | 0 |  |  |  |  |
| The referencing of key statements is inconsistent | 1 |  |  |  |  |
| Key statements are supported by references | 2 |  |  |  |  |
| Scientific reasoning | | 2 | 2 | 2 | 1 |
| The article’s point is not based on appropriate arguments | 0 |  |  |  |  |
| Appropriate evidence is introduced selectively | 1 |  |  |  |  |
| Appropriate evidence is generally presented | 2 |  |  |  |  |
| Appropriate presentation of data | | 2 | 2 | 2 | 2 |
| Data are presented inadequately | 0 |  |  |  |  |
| Data are often not presented in the most appropriate way | 1 |  |  |  |  |
| Relevant outcome data are generally presented appropriately | 2 |  |  |  |  |
| **Sum score** | | 9 | 9 | 8 | 6 |

| **Table 2. Assessment of methodological limitations with the instrument Mixed Methods Approach Tool (MMAT)** | | | | | | | | | | | | | | | | | | | | | | | | | | | | | | | | | | |
| --- | --- | --- | --- | --- | --- | --- | --- | --- | --- | --- | --- | --- | --- | --- | --- | --- | --- | --- | --- | --- | --- | --- | --- | --- | --- | --- | --- | --- | --- | --- | --- | --- | --- | --- |
|  | Methodological quality criteria | **Shaw 2016** | | | **Shaw 2018** | | | **Van den Hurk 2019** | | | **Bitto 2020** | | | **Doughterty 1996** | | | **Massey 2014** | | | **Randall 2005** | | | **Peerbooms 2015** | | | **Lemieux 2014** | | | **Mools 2009** | | | **Van der Hurk 2010** | | |
|  |  | **Responses** | | | **Responses** | | | **Responses** | | | **Responses** | | | **Responses** | | | **Responses** | | | **Responses** | | | **Responses** | | | **Responses** | | | **Responses** | | | **Responses** | | |
|  |  | **Y** | **N** | **CT** | **Y** | **N** | **CT** | **Y** | **N** | **C**  **T** | **Y** | **N** | **CT** | **Y** | **N** | **CT** | **Y** | **N** | **CT** | **Y** | **N** | **CT** | **Y** | **N** | **CT** | **Y** | **N** | **CT** | **Y** | **N** | **C**  **T** | **Y** | **N** | **CT** |
| Screening questions (for all types) | S1. Are there clear research questions? | X |  |  | X |  |  | X |  |  |  |  |  | X |  |  | X |  |  | X |  |  | X |  |  | X |  |  | X |  |  | X |  |  |
|  | S2. Do the collected data allow to address the research questions? | X |  |  | X |  |  | X |  |  |  |  |  | X |  |  | X |  |  | X |  |  | X |  |  | X |  |  | X |  |  | X |  |  |
|  | Further appraisal may not be feasible or appropriate when the answer is ‘No’ or ‘Can’t tell’ to one or both screening questions. |  |  |  |  |  |  |  |  |  |  |  |  |  |  |  |  |  |  |  |  |  |  |  |  |  |  |  |  |  |  |  |  |  |
| 1. Qualitative | 1.1. Is the qualitative approach appropriate to answer the research question? | X |  |  | X |  |  |  |  |  |  |  |  | X |  |  |  |  |  |  |  |  |  |  |  |  |  |  |  |  |  |  |  |  |
|  | 1.2. Are the qualitative data collection methods adequate to address the research question? | X |  |  | X |  |  |  |  |  |  |  |  | X |  |  |  |  |  |  |  |  |  |  |  |  |  |  |  |  |  |  |  |  |
|  | 1.3. Are the findings adequately derived from the data? | X |  |  | X |  |  |  |  |  |  |  |  |  |  |  |  |  |  |  |  |  |  |  |  |  |  |  |  |  |  |  |  |  |
|  | 1.4. Is the interpretation of results sufficiently substantiated by data? | X |  |  | X |  |  |  |  |  |  |  |  |  |  |  |  |  |  |  |  |  |  |  |  |  |  |  |  |  |  |  |  |  |
|  | 1.5. Is there coherence between qualitative data sources, collection, analysis and interpretation? | X |  |  | X |  |  |  |  |  |  |  |  |  |  |  |  |  |  |  |  |  |  |  |  |  |  |  |  |  |  |  |  |  |
| 2. Quantitative randomized controlled trials | 2.1. Is randomization appropriately performed? |  |  |  |  |  |  |  |  |  |  |  |  |  |  |  |  |  |  |  |  |  |  |  |  |  |  |  |  |  |  |  |  |  |
|  | 2.2. Are the groups comparable at baseline? |  |  |  |  |  |  |  |  |  |  |  |  |  |  |  |  |  |  |  |  |  |  |  |  |  |  |  |  |  |  |  |  |  |
|  | 2.3. Are there complete outcome data? |  |  |  |  |  |  |  |  |  |  |  |  |  |  |  |  |  |  |  |  |  |  |  |  |  |  |  |  |  |  |  |  |  |
|  | 2.4. Are outcome assessors blinded to the intervention provided? |  |  |  |  |  |  |  |  |  |  |  |  |  |  |  |  |  |  |  |  |  |  |  |  |  |  |  |  |  |  |  |  |  |
|  | 2.5 Did the participants adhere to the assigned intervention? |  |  |  |  |  |  |  |  |  |  |  |  |  |  |  |  |  |  |  |  |  |  |  |  |  |  |  |  |  |  |  |  |  |
| 3. Quantitative non- randomized | 3.1. Are the participant’s representative of the target population? |  |  |  |  |  |  |  |  |  |  |  |  |  |  |  |  |  |  |  |  |  |  |  |  |  |  | X |  |  |  |  |  |  |
|  | 3.2. Are measurements appropriate regarding both the outcome and intervention (or exposure)? |  |  |  |  |  |  |  |  |  |  |  |  |  |  |  |  |  |  |  |  |  |  |  |  |  | X |  |  |  |  |  |  |  |
|  | 3.3. Are there complete outcome data? |  |  |  |  |  |  |  |  |  |  |  |  |  |  |  |  |  |  |  |  |  |  |  |  | X |  |  |  |  |  |  |  |  |
|  | 3.4. Are the confounders accounted for in the design and analysis? |  |  |  |  |  |  |  |  |  |  |  |  |  |  |  |  |  |  |  |  |  |  |  |  |  |  | X |  |  |  |  |  |  |
|  | 3.5. During the study period, is the intervention administered (or exposure occurred) as intended? |  |  |  |  |  |  |  |  |  |  |  |  |  |  |  |  |  |  |  |  |  |  |  |  |  |  | X |  |  |  |  |  |  |
| 4. Quantitative  descriptive | 4.1. Is the sampling strategy relevant to address the research question? |  |  |  |  |  |  |  |  |  |  | X |  |  |  |  |  |  |  |  |  |  |  |  | X |  |  |  |  | X |  |  |  | X |
|  | 4.2. Is the sample representative of the target population? |  |  |  |  |  |  |  |  |  | X |  |  |  |  |  |  |  |  |  |  |  |  | X |  |  |  |  |  |  | X | X |  | X |
|  | 4.3. Are the measurements appropriate? |  |  |  |  |  |  |  |  |  | X |  |  |  |  |  |  |  |  |  |  |  | X |  |  |  |  |  | X |  |  | X |  |  |
|  | 4.4. Is the risk of nonresponse bias low? |  |  |  |  |  |  |  |  |  | X |  |  |  |  |  |  |  |  |  |  |  |  | X |  |  |  |  |  |  | X |  |  | X |
|  | 4.5. Is the statistical analysis appropriate to answer the research question? |  |  |  |  |  |  |  |  |  | X |  |  |  |  |  |  |  |  |  |  |  | X |  |  |  |  |  | X |  |  | X |  |  |
| 5. Mixed methods | 5.1. Is there an adequate rationale for using a mixed methods design to address the research question? |  |  |  |  |  |  | X |  |  |  |  |  |  |  |  |  |  |  |  |  | X |  |  |  |  |  |  |  |  |  |  |  |  |
|  | 5.2. Are the different components of the study effectively integrated to answer the research question? |  |  |  |  |  |  |  |  | X |  |  |  |  |  |  |  |  |  |  |  | X |  |  |  |  |  |  |  |  |  |  |  |  |
|  | 5.3. Are the outputs of the integration of qualitative and quantitative components adequately interpreted? |  |  |  |  |  |  | X |  |  |  |  |  |  |  |  |  |  |  | X |  |  |  |  |  |  |  |  |  |  |  |  |  |  |
|  | 5.4. Are divergences and inconsistencies between quantitative and qualitative results adequately addressed? |  |  |  |  |  |  | X |  |  |  |  |  |  |  |  |  |  |  |  | X |  |  |  |  |  |  |  |  |  |  |  |  |  |
|  | 5.5. Do the different components of the study adhere to the quality criteria of each tradition of the methods involved? |  |  |  |  |  |  |  | X |  |  |  |  |  |  |  |  |  |  |  |  | X |  |  |  |  |  |  |  |  |  |  |  |  |
| Quality | | High | | | High | | | Low | | | High | | | Low | | | Low | | | Low | | | Low | | | Low | | | Low | | | Low | | |
| CT: can´t tell; N: no; Y: yes | | | | | | | | | | | | | | | | | | | | | | | | | | | | | | | | | | |
